# Measurement of Stable Carbon Isotope Ratio of Volatile Organic Compounds Using Thermal Desorption‐Gas Chromatography‐Combustion‐Isotope Ratio Mass Spectrometry

**DOI:** 10.1155/ianc/9589502

**Published:** 2026-06-16

**Authors:** Li Yuhong, Lin Xihuang, Lin Cifu, Yin Xijie

**Affiliations:** ^1^ Third Institute of Oceanography, Ministry of Natural Resources, Xiamen, China, mnr.gov.cn

**Keywords:** gas chromatography isotope ratio mass spectrometry, stable carbon isotope ratio, thermal desorption, volatile organic compounds

## Abstract

This paper presents a method combining a thermal desorption (TD) unit with gas chromatography‐combustion‐isotope ratio mass spectrometry (GC‐C‐IRMS) to determine compound specific δ^13^C values of seven VOCs (benzene, toluene, ethylbenzene, m/p‐xylene, o‐Xylene, styrene, and cumene). The main innovation lies in the systematic validation of TD‐GC‐C‐IRMS for atmospheric VOC analysis, which enables reliable δ^13^C determination with minimal isotopic bias. The optimal TD conditions were 300°C and 5 min. Advantages include high precision (SD ≤ 0.4‰), good reproducibility, long‐term sample stability (up to 50 days), and suitability for on‐site air sampling. Limitations are the coverage of only seven compounds and a wider range of VOC species and more complex matrices should be addressed in future work. The method was successfully applied to ambient air samples, and isotopic signatures indicated that VOCs were mainly derived from traffic‐related emissions. This approach provides a robust and practical tool for source identification of atmospheric VOCs and supports source apportionment studies in environmental research.

## 1. Introduction

Volatile organic compounds (VOCs) are crucial precursors of ozone and secondary organic aerosols. Some VOCs exhibit carcinogenic, teratogenic, or mutagenic characteristics [1, 2]. The sources of VOCs are intricate, including vehicle exhausts, gasoline evaporation, solvent use, natural gas emissions, and industrial processes [3]. Various approaches have been adopted to trace the origin of atmospheric contaminants and distinguish the contribution of major VOC emission sources to ambient air [4]. Thermal desorption coupled with gas chromatography‐mass spectrometry (TD‐GC‐MS) has been well established for the detection of VOCs in various air quality assessments, such as in hair salons [5] and e‐cigarettes [6]. However, the composition and concentration of a sample often fail to differentiate between distinct VOC sources in environmental studies of complex matrices [7–9]. As a preprint of this work has previously been published [10], the advancement of isotope ratio studies on atmospheric VOCs, compound‐specific stable isotope analysis (CSIA) also applied in many studies for tracing a variety of organic compounds, opened up new avenues to investigate their sources, photochemical histories, and residence times [11].

Rudolph et al. [12, 13] first established a two‐step enrichment device for VOCs in ambient air. VOCs from air samples were enriched in a tube and then rapidly heated to 200°C. Subsequently, the VOCs are transferred to GC capillary column for separation and then introduced into IRMS for carbon isotope ratio measurement. Turner et al. [8] demonstrated the method of TD‐GC‐combustion‐isotope ratio mass spectrometry (TD‐GC‐C‐IRMS) for measuring the δ^13^C of VOCs in emissions. Vitzthum von Eckstaedt et al. [11] measured δ^13^C values of VOCs using TD‐GC‐C‐IRMS in an alumina industry and found that the average δ^13^C values in stack emission were −22‰ to −31‰, which reflects their organic origins from bauxite. Moreover, Vitzthum von Eckstaedt et al. [14] also investigated δ^2^H and δ^13^C values of VOCs from car exhaust emissions, as well as from combustion experiments of various C3 and C4 plants. The results were essential for differentiating between emission such as car exhaust, biomass combustion, and industrial emissions.

The innovation of this study lies in the systematic validation of a TD pretreatment strategy coupled with GC‐C‐IRMS for compound‐specific carbon isotope analysis of VOCs. The proposed method enables efficient enrichment and accurate δ^13^C determination of VOCs in ambient air without significant isotope fractionation. Additional advantages include analytical precision, sample storage stability, and suitability for on‐site atmospheric sampling. This work provides a reliable and practical approach for characterizing carbon isotope compositions of atmospheric VOCs, which is valuable for source identification and process tracing in environmental research.

## 2. Experimental

### 2.1. Chemicals

A standard mixed solution containing benzene, toluene, ethylbenzene, m/p‐xylene, o‐xylene, styrene, and cumene (1000 μg/mL, dissolved in methanol) was purchased from the China National Reference Material Center. Each compound was diluted into 4 concentration gradients: 5, 10, 50, and 100 μg/mL for direct injection by GC‐C‐IRMS. For TD‐GC‐C‐IRMS analysis, the solution was prepared to a concentration of 400 μg/mL.

### 2.2. Instrumentation and Setup

Compound‐specific δ^13^C analysis of atmospheric VOCs was achieved by coupling two well‐established techniques: TD for sample collection and preconcentration (Jingyi technology limited) and GC‐C‐IRMS for the separation of compounds in the sample mixture and accurate δ^13^C measurement (Trace ultra, Delta V advantage, Thermo Fisher Scientific).

**FIGURE 1:**
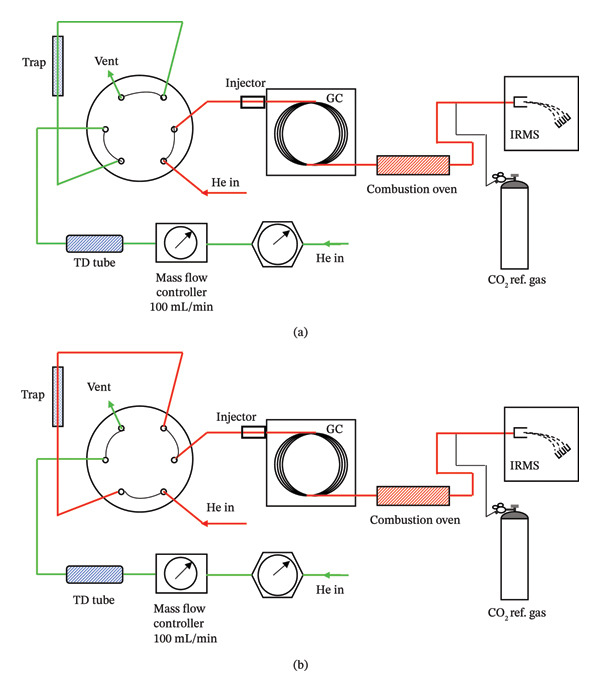
Operational schematic of TD‐GC‐C‐IRMS measurement modes, (a) desorption mode (green line represents the sample gas stream). (b) Injection mode (red line represents the sample gas stream).

TD tubes were stainless steel tubes (O.D. 6.3 mm, I.D. 4.0 mm, and 90 mm length) packed with Tenax TA, conditioned prior to each sampling. During conditioning at 310°C (60 min), a constant pure N_2_ flow (100 mL/min) was passed through them. The cold trap in the TD unit employed a semiconductor cooling device with a stainless steel tube (O.D. 6.3 mm, I.D. 4.0 mm, and 60 mm length) packed with Tenax TA inserted inside. Sample desorption (Figure 1a) was carried out at 300°C and trapped at −30°C for 3 min, where VOCs were trapped and focused, with N_2_ as the carrier gas (50 mL/min). During injection mode (Figure 1b), the trapped VOCs are rapidly desorbed by heating the trap from −30°C to 300°C, transferred to the GC column, separated chromatographically, oxidized in the combustion oven, and detected by IRM. The transfer line temperature was set at 200°C. The GC was equipped with a CD‐VOCOL column (60 × 0.25 mm × 1.8 μm, Agilent J&W) with a column flow of 1.5 mL/min. The temperature program is as follows: isothermal at 40°C for 2 min, ramped to 180°C at 4°C/min, then ramped to 220°C at 20°C/min and held for 2 min. Both direct injection and TD injection adopted the pulsed‐splitless mode (split/splitless injector).

### 2.3. Air Samples

Air samples were drawn through TD tubes using a compact Gilian LFS hand pump, the pump operates at a constant flow. Flexible Tygon tubing was used to connect the TD tube to the pump, no particulate filter such as a glass wool was used. The sampling volume was adjusted according to the concentration in the range of 1–20 L to ensure sufficient VOCs for stable isotope analysis. Subsequently, the TD tube was sealed with a polytetrafluoroethylene cap and stored in an airtight container under refrigerated conditions and analyzed within 30 days. All procedures were carried out following the methods described by Harper [15] and Elizabeth [16].

### 2.4. Analytical Validation

Precision was assessed by repeatability, expressed as standard deviation (SD) of δ^13^C values. Accuracy was evaluated by comparing δ^13^C results between TD injection and direct injection mode. Robustness was verified by examining the effects of TD temperature (280°C–300°C) and TD time (3–5 min), which should cause no significant isotope fractionation. Univariate optimization was performed for qualitative separation of VOCs and quantitative analysis of their carbon isotope ratios.

### 2.5. Statistical Analysis

All measurements were performed in at least triplicate (*n* ≥ 3). Data are presented as mean ± standard deviation (SD). The stability of δ^13^C values under different conditions was evaluated by comparing mean values and SD. Differences between injection methods and storage times were considered insignificant when SD ≤ 0.5‰, which is acceptable for compound‐specific isotope analysis [17].

### 2.6. Isotope Ratio Calculation

The stable isotopic ratio is expressed as δ relative to the reference gas (‰), and the calculation formula is as follows:

(1) δ13C ‰=Rsample−RstandardRstandard×103=RsampleRstandard−1×103,

where the CO_2_ reference gas (−20.70‰) was calibrated to IAEA‐600 (−27.77‰) and NBS‐19 (1.95‰) by an elemental analyzer (Flash EA, Thermo Fisher Scientific) coupled with the same IRMS used in the TD‐GC‐C‐IRMS method.

## 3. Results and Discussion

### 3.1. Method Linearity and Impact of TD Temperature and Time

**FIGURE 2:**
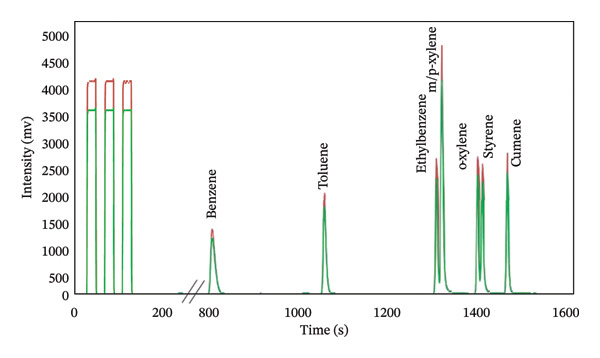
IRMS chromatogram of the VOC standard mixture by the TD‐GC‐C‐IRMS method in this study.

As the Delta series IRMS from Thermo Fisher recommends a minimum sample amount of at least 1 nmol or 10 ng carbon, the detection limits of each injection device were examined. An example of a typical chromatogram measured by the IRMS was shown in (Figure 2). Baseline peak separation is generally desirable in GC‐C‐IRMS to minimize isotopic bias from co‐elution, especially for closely eluting compounds such as ethylbenzene and m/p‐xylene or o‐xylene and styrene. Although individual and mixed standards were not compared in this work, the consistent δ^13^C results and low uncertainty across replicates indicate that the achieved chromatographic resolution was sufficient for reliable isotopic analysis.

**FIGURE 3:**
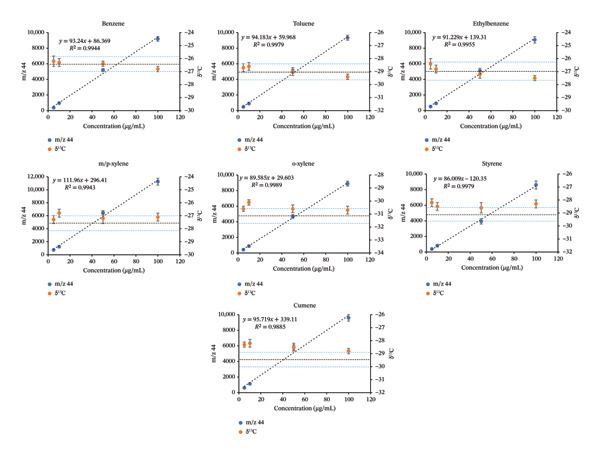
Linear correlations of δ^13^C values and m/z = 44 signal intensity with varying concentrations for the 7 compounds (the black dashed line indicates the average value, while the blue dashed line denotes a ±0.5‰ deviation).

Seven benzene series standards were diluted into concentration gradients of 5, 10, 50, and 100 μg/mL, with triplicate injections for each concentration (injection volume of 1 μL) to investigate the effect of sample amount on isotopic fractionation of each component. As shown in (Figure 3), for the seven compounds, a good linear relationship in the range from approximately 500 to 10,000 mV (*R* ^2^ > 0.99) was observed. The signal intensity reached approximately 500 mV at a sample amount of 5 μg/mL, and the isotopic values of each component remained stable. Therefore, in actual sample analysis, each component should be enriched to a signal intensity above 500 mV.

When using the TD unit for sample analysis, TD temperature and time are the main factors that influence the efficiency of the experiments; possible isotopic fractionation during this process should be evaluated. This test was achieved using a TD tube spiked with a standard solution (400 μg/mL, 1 μL) with a number of TD temperatures and TD times, ranging from 280°C to 300°C, 3–5 min, respectively. It was established that δ^13^C of 7 compounds were consistent and within the error of analysis. The results show that the different TD temperature and time cause no significant fractionation of the compounds. Therefore, 300°C and 5 min were selected as the optimal conditions to ensure complete desorption of analytes from the tubes.

### 3.2. Effect of Injection Pattern on Isotope Fractionation

Replicate analysis was performed using the same volume of standard solution (400 μg/mL, 1 μL) and identical TD method to investigate method reproducibility. Meanwhile, the δ^13^C values obtained by direct injection and TD were compared to assess whether isotope fractionation occurred. Table 1 shows δ^13^C and standard error of analysis using both methods. The difference in measured δ^13^C values between the two methods is less than 0.2%, and the standard deviation varied from 0.1% to 0.4%. In comparison with previous studies [9, 10, 13, 14], the analytical precision and intermethod differences in this study were reasonable, confirming that TD‐GC‐C‐IRMS is suitable for measuring δ^13^C of atmospheric VOCs.

**TABLE 1:** Comparison of isotopic signatures (δ^13^C, ‰) and standard errors for 7 compounds via direct injection and TD method.

Injection mode	Direct injection (μg/mL)						TD δ^13^C ± SD (*n* = 8)
	δ^13^C ± SD (*n* = 3)						
Compound	5	10	50	100	500	Total (*n* = 15)	
Benzene	−26.2 ± 0.4	−26.3 ± 0.2	−26.4 ± 0.1	−26.8 ± 0.1	−27.1 ± 0.1	−26.6 ± 0.3	−26.7 ± 0.4
Toluene	−28.7 ± 0.2	−28.6 ± 0.2	−29.0 ± 0.1	−29.4 ± 0.2	−29.2 ± 0.1	−29.0 ± 0.2	−29.2 ± 0.4
Ethylbenzene	−26.4 ± 0.4	−26.8 ± 0.2	−27.2 ± 0.3	−27.5 ± 0.2	−27.6 ± 0.1	−27.1 ± 0.2	−27.2 ± 0.4
m/p‐Xylene	−27.3 ± 0.2	−26.8 ± 0.2	−27.2 ± 0.3	−27.1 ± 0.1	−27.0 ± 0.1	−27.1 ± 0.2	−27.0 ± 0.2
o‐Xylene	−30.6 ± 0.1	−30.1 ± 0.1	−30.6 ± 0.3	−30.7 ± 0.2	−30.5 ± 0.1	−30.5 ± 0.1	−30.4 ± 0.3
Styrene	−28.2 ± 0.4	−28.5 ± 0.2	−28.6 ± 0.3	−28.3 ± 0.3	−28.0 ± 0.2	−28.3 ± 0.2	−28.2 ± 0.3
Cumene	−28.3 ± 0.2	−28.2 ± 0.3	−28.5 ± 0.2	−28.8 ± 0.2	−29.1 ± 0.1	−28.6 ± 0.2	−28.7 ± 0.4

### 3.3. Effect of Storage Time on δ^13^C

Few experiments were applied to evaluate δ^13^C variation during the storage [10]. Standard TD tubes in this study were prepared in accordance to the method from [1]. A 1 μL aliquot of the standard mixture (400 μg/mL) was injected directly into the TD tubes, followed by purging with high‐purity N_2_ through the tubes for 3 min at 50 mL/min. All TD tubes were stored at 4°C in an airtight bag containing activated charcoal. After approximately 50 days of storage, the δ^13^C values in Table 2 shows negligible differences (SD between 0.1‰ and 0.4‰) for 7 compounds.

**TABLE 2:** Results of δ^13^C (‰) analyses in the TD tubes with 7 compounds approximately 50 days of storage.

Time of storage (d)	1	15	24	35	48	Average	SD
Benzene	−26.3	−27.3	−26.8	−27.1	−26.4	−26.9	0.4
Toluene	−28.6	−29.7	−29.4	−29.6	−29.0	−29.3	0.5
Ethylbenzene	−26.8	−27.5	−27.5	−27.6	−27.2	−27.4	0.3
m/p‐Xylene	−26.8	−26.9	−27.1	−27.0	−27.2	−27.0	0.1
o‐Xylene	−30.1	−30.6	−30.7	−30.5	−30.6	−30.5	0.3
Styrene	−28.5	−27.9	−28.3	−28.0	−28.6	−28.1	0.3
Cumene	−28.2	−29.1	−28.8	−29.1	−28.5	−28.8	0.4

### 3.4. Analysis of Air Samples

Six replicate air samples were analyzed, and 5 components were identified based on retention time of standards. The δ^13^C values ranged from −24.7‰ (benzene) to −30.3‰ (ethylbenzene) (Table 3). δ^13^C data of VOCs from typical pollution sources are very limited. Wang et al. [18] measured δ^13^C of volatile gasoline, vehicle exhaust, volatile diesel, and diesel exhaust sources; it was found that δ^13^C from air samples has similar values to those from vehicle and gas station emissions. The value for benzene is closer to those from the vehicle and diesel exhaust, and the value for ethylbenzene resembles those from volatile gasoline. Given that the ambient air sampling point was near a parking area and expressway, the results reasonably indicate a significant contribution of traffic sources to local VOCs.

**TABLE 3:** δ^13^C (‰) of 5 VOCs in measurements of air samples (*n* = 6) and values from several typical sources of contamination in previous research.

Compounds	Average (this study)	SD	Volatile gasoline [18]	Vehicle exhaust [16]	Volatile diesel [16]	Diesel exhaust [18]
Benzene	−24.7	0.2	−28.0	−23.9	−26.0	−25.4
Toluene	−25.1	0.6	−29.5	−27.9	−26.4	−23.9
Ethylbenzene	−30.3	0.9	−30.9	−27.4	−26.5	−26.0
m/p‐Xylene	−28.1	0.4	−32.8	−32.3	−28.7	−32.9
o‐Xylene	−27.2	0.4	−29.6	−27.7	−23.7	−25.4

In summary, this study systematically validated the performance of TD‐GC‐C‐IRMS for determining compound‐specific δ^13^C values of VOCs. When applied to ambient air samples, the measured δ^13^C signatures effectively supported source identification and indicated significant contributions from traffic‐related emissions. These findings demonstrate that the optimized TD‐GC‐C‐IRMS approach is robust and suitable for routine δ^13^C analysis of atmospheric VOCs, providing reliable support for source tracing in atmospheric environmental research.

## 4. Conclusion

This study established a TD‐GC‐C‐IRMS method for compound‐specific stable carbon isotope analysis of VOCs. The innovation of this work lied in the systematic validation of a TD‐based preconcentration strategy coupled with GC‐C‐IRMS, enabling reliable δ^13^C determination of atmospheric VOCs without significant isotope fractionation.

The advantages include high precision (SD ≤ 0.4‰), good reproducibility, long‐term sample stability (stable for about 50 days), and suitability for on‐site air sampling. The method yields δ^13^C results comparable to direct injection and supports accurate source identification in real ambient samples. However, several limitations should be noted. First, the method focuses on seven target VOCs and is not yet validated for other VOC species. Second, the quantification and isotope accuracy rely on sufficient peak separation, which requires careful control of GC conditions.

Overall, the validated TD‐GC‐C‐IRMS method provides a reliable tool for characterizing δ^13^C values of atmospheric VOCs. It strengthens traditional VOC monitoring approaches and supports better understanding of VOC sources, atmospheric processes, and pollution control strategies.

## Author Contributions

Li Yuhong: methodology, investigation, experiment, and writing–review and editing. Lin Xihuang: instrument maintenance and experiment. Lin Cifu: investigation and experiment. Yin Xijie: resources, supervision, and funding.

## Funding

This study was sponsored by the Scientific Research Foundation of Third Institute of Oceanography (2024014) and Natural Science Foundation of China (42403052).

## Conflicts of Interest

The authors declare no conflicts of interest.

## Data Availability

The data that support the findings of this study are available from the corresponding author upon reasonable request.
